# Rapid CD4 decline after interruption of non-nucleoside reverse transcriptase inhibitor-based antiretroviral therapy in a resource-limited setting

**DOI:** 10.1186/1742-6405-4-26

**Published:** 2007-11-21

**Authors:** Somnuek Sungkanuparph, Sasisopin Kiertiburanakul, Anucha Apisarnthanarak, Kumthorn Malathum, Siriorn Watcharananan, Boonmee Sathapatayavongs

**Affiliations:** 1 Faculty of Medicine Ramathibodi Hospital, Mahidol University, Bangkok, Thailand; 2 Faculty of Medicine, Thammasart University Hospital, Pratumthani, Thailand

## Abstract

**Background:**

Non-nucleoside reverse transcriptase inhibitor (NNRTI) with stavudine and lamivudine is widely used as the first-line antiretroviral therapy (ART) in resource-limited settings. Lipodystrophy is common and options for switching ART regimen are limited; this situation can lead to patients' poor adherence and antiretroviral resistance. Treatment interruption (TI) in patients with high CD4 cell counts, lipodystrophy, and limited options may be an alternative in resource-limited settings. This study aimed to determine time to resume ART after TI and predictors for early resumption of ART in a resource-limited setting.

**Methods:**

A prospective study was conducted in January 2005 to December 2006 and enrolled HIV-infected patients with HIV-1 RNA <50 copies/mL, CD4 > 350 cells/mm^3^, and willing to interrupt ART. CD4 cell count, HIV-1 RNA, lipid profile, and lipodystrophy were assessed at baseline and every 3 months. ART was resumed when CD4 declined to <250 cells/mm^3 ^or developed HIV-related symptoms. Patients were grouped based on ART regimens [NNRTI or protease inhibitor (PI)] prior to TI.

**Results:**

There were 99 patients, 85 in NNRTI group and 14 in PI group. Mean age was 40.6 years; 46% were males. Median duration of ART was 47 months. Median nadir CD4 and baseline CD4 were 151 and 535 cells/mm^3^, respectively. Median CD4 change at 3 months after TI were -259 (NNRTI) and -105 (PI) cells/mm^3 ^(p = 0.038). At 13-month median follow-up, there was no AIDS-defining illness; 38% (NNRTI) and 29% (PI) of patients developed HIV-related symptoms. ART was resumed in 51% (NNRTI) and 36% (PI) of patients (p = 0.022). By Kaplan-Meier analysis, median time to resume ART was 5.5 (NNRTI) and 14.2 (PI) months (log rank test, p = 0.026). By Cox's regression analysis, NNRTI-based ART (HR 4.9; 95%CI, 1.5–16.3), nadir CD4 <100 cells/mm^3 ^(HR 2.7; 95%CI 1.4–5.3) and baseline CD4 <500 cells/mm^3 ^(HR 1.6; 95%CI, 1.2–3.1) were predictors for early ART resumption.

**Conclusion:**

TI of NNRTI-based ART leads to rapid CD4 decline and high probability of early ART resumption and should be avoided. It is necessary to scale-up the options for HIV-infected patients with lipodystrophy in resource-limited settings.

## Background

Highly active antiretroviral therapy (HAART) has dramatically changed the course of human immunodeficiency virus type 1 (HIV-1) disease, with a substantial reduction in morbidity and mortality [1-3]. New antiretroviral drugs and combinations with better safety and tolerability profiles have become available in developed countries [4,5], but these options are still not available or are not affordable in resource-limited settings. Non-nucleoside reverse transcriptase inhibitor (NNRTI) with stavudine and lamivudine is widely used as the first-line antiretroviral therapy (ART) in resource-limited settings [6,7]. Lipodystrophy is common and the options for switching ART regimen are limited; this situation can lead to patient's poor adherence on ART and subsequent antiretroviral resistance [8,9]. Treatment interruption (TI) in patients with high CD4 cell counts, lipodystrophy, and limited options may be an alternative in resource-limited settings.

Prior to the publcation of the Strategy for Management of Antiretroviral Therapy (SMART) study [10], several studies had been testing the strategy of using CD4 cell count to guide when to interrupt and recommence ART [11-14]. However, there is none study reporting the difference outcomes of TI between NNRTI-based and protease inhibitor (PI)-based ART. From Staccato study [12], types of regimens were not associated with disease progression or time to resume ART. However, most study patients in Staccato study received PI-based ART. This study aimed to determine time to resume ART after TI of NNRTI-based ART and evaluate the predictors for early resumption of ART in a resource-limited setting.

## Methods

A prospective study was conducted in HIV-1-infected patients who had high CD4 cell counts and complete HIV-1 suppression (<50 copies/mL) at a medical-school hospital. Participants were enrolled between January 2005 and December 2005 and were followed through the end of December 2006. Inclusion criteria were as follows: 1) HIV-1-infected patients > 15 years of age, 2) receiving an NNRTI-based or PI-based ART as an initial regimen, 3) had undetectable HIV-1 RNA (<50 copies/mL), 4) had CD4 cell count >350 cells/mm^3^, and 4) willing to interrupt ART. All patients continued dual NRTIs for a further 7-day duration after TI of nevirapine-based regimens and a 10-day duration for efavirenz-based regimens. Lipid lowering agents were continued in patients who had been receiving these drugs prior to participate in this study.

CD4 cell count, HIV-1 RNA, glucose and lipid profile including total cholesterol (TC), LDL-C, HDL-C, and triglycerides (TG) were monitored at baseline and in every 3 months. Lipodystrophy was defined by a change in body fat distribution reported by the patients and assessed by the same investigator (SS) who was trained for this assessment at baseline and in every 3-month clinic visit.

ART was resumed when CD4 cell count declined to <250 cell/mm^3 ^or developed HIV-related symptoms. After report of the SMART study in November 2006, the participated patients were notified the results of SMART study and decided to resume ART or continued TI with closed follow-up. CD4 cell count was monitored every 6 weeks in patients who decided to continue TI. Patients were grouped based on their ART regimens prior to TI, NNRTI-based regimens (NNRTI group) or PI-based regimens (PI group). The study was approved by the Institutional Review Board and written informed consent was obtained from all participants.

The primary objective of the study was to determine the time to resume ART after TI of NNRTI-based regimens. The secondary objectives were to: i) compare time to resume ART after TI between NNRTI group and PI group, ii) define the predictors for early resumption of ART, iii) compare change of CD4 and HIV-related symptoms after TI between NNRTI group and PI group, and iv) determine changes of lipid profile and lipodystrophy after TI.

Median (interquatile range, IQR) and frequencies (%) were used to describe patients' characteristics in both groups. Chi-square (or Fisher exact test where appropriate) and Mann-Whitney U tests were used to compare categorical and continuous variables between the two study groups, respectively. The Kaplan-Meier test was used to estimate the median time to resume ART between the two groups. The patients were censored when they resumed ART or at the end of study. Log-rank test was used to compared the median time to resume ART between groups. Statistical calculations were performed using SPSS program version 13.0 (SPSS Inc., Chicago, Illinois, U.S.A). A two-sided *P *value of less than 0.05 was considered statistically significant.

## Results

**Figure 1:**
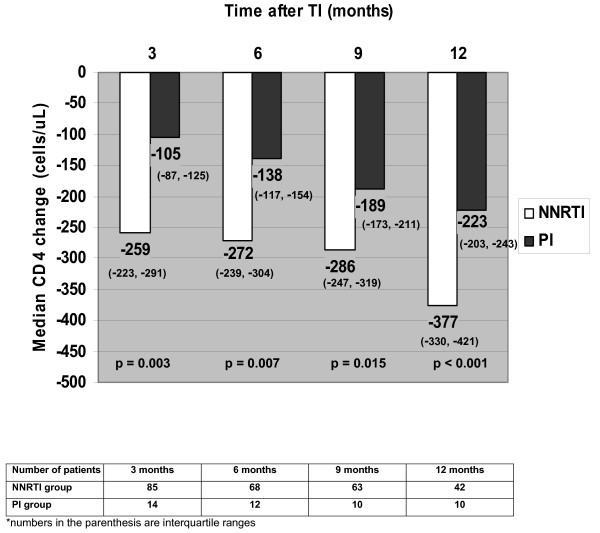
Changes of median CD4 cell counts after TI in NNRTI and PI group.

A total of 99 patients participated in this study, 85 (86%) patients in NNRTI group and 14 (14%) patients in PI group. The mean (SD) age was 40.6 (9.1) years old and 46% were males. Baseline characteristics of patients in both groups are shown in Table 1. Of all patients, 83% had lipodystrophy. After TI, median HIV-1 RNA levels were rapidly increased from <1.7 log copies/mL at baseline to 4.8, 5.0, 4.8, and 4.7 log copies/mL at 3, 6, 9, and 12 months, respectively. There were no differences of HIV-1 RNA levels between the two groups at each time point (p > 0.05). Change of median CD4 cell counts at 3, 6, 9, and 12 months after TI are demonstrated in Figure 1. At a median follow-up duration of 13 months, there was no AIDS-defining illness; 32 (38%) patients in NNRTI group and 4 (29%) patients in PI group developed HIV-related symptoms. The symptoms included weight loss (58%), fever (17%), pruritic papular eruption (11%), oral candidiasis (8%), and diarrhea (6%). ART was resumed in 43 (51%) patients in NNRTI group and 5 (36%) patients in PI group (p = 0.022). Reasons of ART resumption were as follows: CD4 <250 cells/mm^3 ^(79%), developed symptoms (18%), and patients' decision (3%).

**Table 1:** Clinical characteristics of patients in NNRTI and PI group.

Characteristics	NNRTI *N = 85*	PI *N = 14*	P-value
Age, mean ± SD, years	40.6 ± 8.8	40.7 ± 10.9	0.776
Gender, number (%)			0.993
Male	38 (45)	7 (50)	
Female	47 (55)	7 (50)	
Duration of HIV diagnosis, median (IQR), months	65 (47–94)	72 (47–109)	0.788
History of OIs, number (%)	10 (12)	2 (14)	0.992
HBV co-infection, number (%)	6 (7)	1 (7)	0.957
HCV co-infection, number (%)	3 (4)	0 (0)	0.451
NRTI backbone in HAART prior to TI, number (%)			0.480
- Stavudine + lamivudine	43 (51)	7 (50)	
- Zidovudine + lamivudine	35 (41)	5 (36)	
- Zidovudine + didanosine	4 (5)	2 (14)	
- Didanosine + lamivudine	3 (3)	0 (0)	
Duration of HAART prior to TI, median (IQR), months	46 (36–64)	59 (37–76)	0.968
Nadir CD4 cell count, median (IQR), cells/mm^3^	147 (57–215)	217 (63–345)	0.186
Baseline CD4 cell counts at TI, median (IQR), cells/mm^3^	530 (441–657)	586 (381–747)	0.924

OIs = opportunistic infections, HBV = hepatitis B virus, HCV = hepatitis C virus

**Figure 2:**
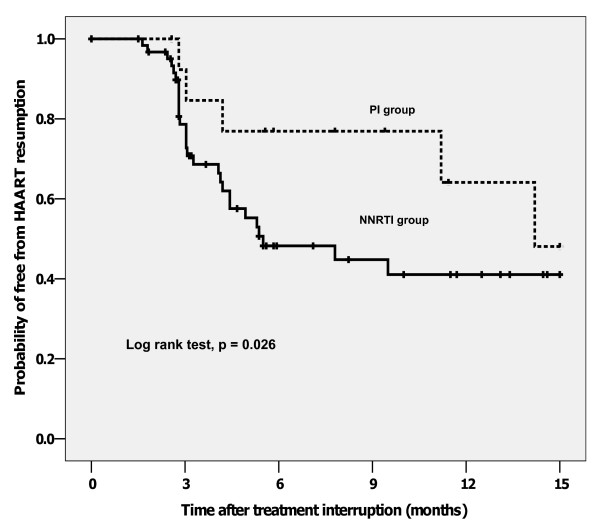
Kaplan-Meier analysis for the probability of free from ART resumption.

By Kaplan-Meier analysis, median time to resume ART was 5.6 months in NNRTI group and 15.0 months in PI group (log rank test, p = 0.026, Figure 2). By Cox's regression, NNRTI-based ART [hazard ratio (HR) 4.9; 95% confidence interval (CI), 1.5–16.3], nadir CD4 <100 cells/mm^3 ^[HR 2.7; 95%CI 1.4–5.3] and baseline CD4 <500 cells/mm^3 ^[HR 1.6; 95%CI 1.2–3.1] were predictors for early ART resumption. Duration of ART was not associated with early ART assumption.

Among 51 patients who did not need ART resumption after TI for >12 months, there was a significant decrease of TG at 12 months when compared to baseline (165 vs. 247 mg/dL, p = 0.012). In contrast, there were no significant differences of TC (208 vs. 232 mg/dL, p = 0.062), LDL-C (143 vs. 145 mg/dL, p = 0.521), and HDL-C (33 vs. 46 mg/dL, p = 0.055) from baseline. Only two patients had high fasting plasma glucose at baseline and there was no significant change of mean plasma glucose after TI. Of 82 patients who had lipodystrophy at baseline, five (6%) patients had improved lipodystrophy. All these five patients had TI >12 months.

## Discussion

The primary results from the present study has demonstrated that TI of NNRTI-based regimens is associated with a rapid CD4 decline when compared to PI-based regimens. When compared to the results from SMART study [10], the overall rate of CD4 decline was comparable whereas this rate in NNRTI group was more rapidly declined. This results in the need for early resumption of ART in patients who had TI of NNRTI-based ART. Previous CD4 cell count-guided studies suggest that CD4-guided TI may permit safe TI without major clinical complications in HIV-infected patients with complete viral suppression [11-19]. In contrast, the large SMART study have found that patients with CD4-guided TI are at a significantly higher risk of severe clinical events and death than those with continuing ART [10]. This finding prompts many on-going CD4-guided TI studies including the present study to close trials. Nevertheless, recent ACTG 5170 study [11] and Staccato study [12] have addressed that CD4-guided TI may be safe in some specific groups.

Interestingly, Staccato [12] and TRIVACAN [20] studies has different outcomes of CD4-guided TI. Both studies have similar number of study patients. In addition to the fact that these two studies resumed ART at different CD4 levels (< 350 cells/mm^3 ^in Staccato and < 250 cells/mm^3 ^in TRIVACAN), one major difference between these two studies is ART regimen in study patients; 80% of patients in Staccato received PI-based regimens whereas 90% of patients in TRIVACAN study had NNRTI-based regimens. Although SMART and TIBET [21] study included both NNRTI- and PI-based ART, there were no analyses to determine the outcomes of TI between NNRTI- and PI-based regimens. The results from the present study herein addresses this issue; TI of NNRTI-based ART is 5-time more likely to need early ART resumption after TI, when compared to TI of PI-based ART. ACTG 5142 study has recently reported that PI-based ART yielded a better immunological response than NNRTI-based ART [22]. This may indirectly explain the results from the present study.

We also found that nadir CD4 <100 cells/mm^3 ^and baseline CD4 <500 cells/mm^3 ^were significant predictors for early ART resumption. These findings were concordant with the results from previous studies [11,13-15,17,21]. Although CD4 rapidly declined in NNRTI group, we found that there was no difference of viral rebound after TI. In addition, HIV-1 RNA was abruptly increased after TI. This was concordant with the high incidence of HIV-related symptoms in the present study. The further analysis (data not shown) did not show any correlation between rate of HIV-1 RNA rising and early ART resumption. The results of improved TG after TI may be some benefits from TI. However, the potential risks do outweigh these benefits. Although some patients had improved lipodystrophy particularly when their durations of TI were long enough, i.e. >12 months. However, TI is not a good solution for lipodystrophy because TI of NNRTI-based regimens has a rapid CD4 decline and a high probability of early ART resumption.

In resource-limited settings where NNRTI with stavudine and lamivudine is widely used as the first-line ART, using stavudine in first-line ART should be reconsidered. Given a large amount of patients in developing countries currently receive a regimen of stavudine, lamivudine, and nevirapine, it is necessary to scale-up the options for HIV-infected patients who develop lipodystrophy in resource-limited settings. National ART access program in developing countries is needed to be better prepared.

The present study has some limitations. First, the study was small according to the limited budget. The second phase of study was not granted after the report of SMART study. Second, the proportion of patients in PI group was much smaller than that of NNRTI group. This could be explained by the fact that the majority of patients in developing countries taking NNRTI-based ART. However, the sample size of the present study was enough to demonstrate the different outcomes of TI from NNRTI-based and PI-based ART. Third, there was a high proportion of patients with lipodystrophy in the present study. According to the inclusion criteria that we enrolled patients who were willing to have TI, those with lipodystrophy were more likely to participate in the study. The results of improved TG levels in the present study may not be applicable for other population with a lower prevalence of lipodystrophy and dyslipidemia.

In conclusions, TI of NNRTI-based ART leads to rapid CD4 decline and the need for early ART resumption. TI is not a safe alternative for patients with lipodystrophy and limited options in resource-limited settings and, therefore, should be avoided. It is necessary to scale-up the options for HIV-infected patients with lipodystrophy in resource-limited settings. Other strategies to manage with limited resources, as well as reconsideration of using stavudine in the first-line ART regimen in developing countries, should be evaluated.

## Abbreviations

ART: Antiretroviral therapy;

HAART: Highly active antiretroviral therapy; 

HIV: Human immunodeficiency virus; 

NNRTI: Non-nucleoside reverse transcriptase inhibitors. 

## Competing interests

The author(s) declare that they have no competing interests.

## Authors' contributions

SS participated in the design of the study, clinical assessment of study patients, performed statistical analysis, and drafting the manuscript. SK participated in clinical assessment of patients and drafting the manuscript. AA participated in drafting the manuscript. KM participated in clinical assessment of study patients. SW participated in clinical assessment of patients and drafting the manuscript. BS participated in clinical assessment of patients and drafting the manuscript. All authors read and approved the final manuscript.
